# Boosting low-resolution algae detection via cross-resolution knowledge distillation and transfer learning

**DOI:** 10.3389/fmicb.2026.1914806

**Published:** 2026-08-28

**Authors:** Mingjie Jiang, Mingxia Yang, Yuxun Wu, Haixia Zheng

**Affiliations:** 1 College of Electrical and Information Engineering, Quzhou University, Quzhou, Zhejiang, China; 2 School of Computing, University of Portsmouth, Portsmouth, United Kingdom

**Keywords:** deep learning, harmful algae detection, knowledge distillation, object detection, transfer learning

## Abstract

**Objective:**

To address the critical challenge of degraded detection performance caused by low-resolution microscopic inputs in field-based harmful algae monitoring, this paper proposes a cross-resolution knowledge distillation framework integrated with transfer learning. The method transfers fine-grained feature representations from a high-resolution YOLO teacher model to a low-resolution YOLO student model, thereby enabling accurate and efficient harmful algae identification at no additional inference cost.

**Methods:**

Built upon the EMDS-7 environmental microorganism dataset, we employ a YOLO student network with a low input resolution, initialized with COCO pre-trained weights. A YOLO teacher model, trained at 1280 × 1,280 resolution, provides high-resolution feature supervision. To transfer the fine-grained spatial cues, we design a multi-scale attention-based distillation loss that aligns the spatial attention maps between student and teacher at the P3, P4, and P5 neck levels. The overall training objective is a weighted sum of the original detection loss and the feature distillation loss.

**Results:**

On the test set of EMDS-7, the proposed method achieves a mAP50-95 of 0.78832 and a mAP50 of 0.87897, outperforming the undistilled baseline (0.78434 and 0.87341) as well as other methods such as YOLOv5s, YOLOv8s, YOLOv10s, YOLOv12s and YOLOv26s. Precision and recall reach 0.87542 and 0.84143, respectively. Ablation studies on the distillation weight λ show that λ=0.5 gives the best performance, with lower or higher values leading to degraded results.

**Conclusion:**

The proposed cross-resolution knowledge distillation framework effectively mitigates the fine-grained feature degradation inherent in low-resolution algae imagery, while transfer learning provides the student model with robust visual priors. The resulting lightweight detector, operating at low resolution, is well-suited for deployment on field-portable microscopic platforms. This work offers a viable pathway toward intelligent harmful algae monitoring in resource-constrained field settings.

## 1 Introduction

Aquatic ecosystems are a vital component of the Earth’s biosphere. Harmful algae, as key microbial groups in water bodies, can undergo abnormal proliferation and trigger algal blooms, which have become a global water-environmental hazard (Wang et al., 2026). The global incidence of harmful algal blooms has increased since the 1980s, with a 44% surge from the 2000s to the 2010s, and the most pronounced effects were observed in Asia and Africa (Feng et al., 2024). When harmful algae break out on a large scale, they not only deplete dissolved oxygen and destroy aquatic habitats, leading to reduced aquaculture yields, but certain species also release algal toxins (Brown et al., 2020). Therefore, achieving rapid and accurate classification and identification of harmful algae is a fundamental prerequisite for water quality monitoring, algal bloom early warning, and aquatic ecological restoration. Traditional algae identification mainly comprises four technical systems: morphological structure-based detection, cytochrome-based techniques, immunoassays, and nucleic acid-based detection (Liu et al., 2022). However, these methods suffer from low efficiency, susceptibility to human error, and an inability to achieve batch monitoring.

With the advancement of computer vision, deep learning has become the mainstream direction for algae identification, with related research divided into two branches: image classification and object detection. Many studies have been conducted using classical convolutional neural networks in algae image classification domain. Py et al. (2016) proposed a deep neural network that leverages translational and rotational symmetry, with constrained CNN designs and an Inception-like structure for multi-size inputs, and demonstrates its feasibility on the Plankton Set 1.0 dataset. Khaldi and Khaldi (2024) evaluated state-of-the-art CNN models with transfer learning for classifying harmful phytoplankton, achieving 96.97% accuracy with ResNet-50 via fine-tuning. Sonmez et al. (2024) applied convolutional neural networks and support vector machines with linear kernel to achieve high classification accuracy, far surpassing slow, expert-dependent traditional methods. Tian and Mao (2025) developed an automated red-tide algae classification system using an improved Canny edge detector for cell extraction and a lightweight Vision Transformer. In algae detection domain, YOLO-series single-stage object detection algorithms, with their advantages of end-to-end inference and a good balance between speed and accuracy, have become mainstream solutions for microscopic image detection and have been extensively used in plankton and algae identification scenarios. An et al. (2025) introduced an intelligent planktonic algae recognition system based on the YOLOv5x model, which achieves 63.7% mAP50 across 55 species, enabling automated whole-slide analysis for rapid detection and early warning of harmful algal blooms. Taheriashtiani et al. (2026) presented a deep-learning workflow using YOLOv12, achieving strong in-domain accuracy but revealing severe cross-region generalization drops, though merging data from multiple regions partly recovers performance. Wu et al. (2026) proposed AlgaeFusion-YOLO, a semantic-augmented feature fusion framework built on YOLO11 that incorporates WTConv, CBAM (Woo et al., 2018), BiFPN (Tan et al., 2020), and few-shot learning with CLIP supervision to address feature extraction difficulties and class imbalance, achieving high mAP on an algal dataset. Yang et al. (2023) built EMDS-7 dataset which is a microscopic image dataset designed for AI-based multi-object detection, containing 41 environmental microorganism types. The dataset was validated using Faster-RCNN (Girshick, 2015), YOLOv3 (Redmon and Farhadi, 2018), YOLOv4 (Bochkovskiy et al., 2020), SSD (Kumar et al., 2023), and RetinaNet (Lin et al., 2017). Guo et al. (2026) evaluated 25 object detectors on the EMDS-7 dataset, revealing that two-stage methods like Faster R-CNN achieve top accuracy, while precise localization remains the primary bottleneck and deeper backbones offer limited gains using from-scratch training. Dong et al. (2024) integrated a lightweight C2F Faster backbone, Focal-SIoU loss, and SRGAN-based data augmentation into YOLOv8s to improve the performance while reducing parameters and FLOPs. Beyond its success in algae detection, YOLO has also been adopted as a core component in other vision tasks, such as tumor and lesion detection (Kang et al., 2025), pedestrian trajectory prediction (Jiang et al., 2026), object detection in remote sensing (Liu S. et al., 2026).

However, existing algae detection models still face the challenge of image resolution discrepancy. Low-resolution object detection has also attracted increasing attention in recent years. Methods such as HFA-YOLO have been specifically designed for recognizing low-resolution small targets on water surfaces (Liu T. et al., 2026), while RT-FCOSH addresses the accuracy-efficiency trade-off in low-resolution autonomous driving scenarios (Mboutayeb et al., 2026). These efforts underscore the growing recognition that low-resolution input is a hard constraint in many real-world deployment scenarios, rather than an optional trade-off.

Portable microscopic devices in the field, limited by hardware, often capture low-resolution algae images with blurred details and missing edge features; meanwhile, high-resolution image samples obtained from high-precision laboratory equipment cannot be directly adapted to low-resolution detection scenarios, significantly degrading the model’s practical deployment performance. Model distillation, via a teacher–student architecture, transfers feature representation capabilities from a high-precision complex model to a lightweight one, balancing detection accuracy and deployment efficiency (Hu et al., 2023). Recent surveys have systematically reviewed distillation methods for object detection, covering both CNN-based and Transformer-based detectors (Shehzadi et al., 2026; Golizadeh et al., 2025). Multi-dimensional knowledge distillation strategies—incorporating local feature distillation, attention distillation, and global feature distillation—have been proposed for one-stage detectors (Li et al., 2024). Hierarchical matching knowledge distillation, which distills knowledge from P2 to P4 pyramid levels, was developed specifically for small object detection to preserve fine-grained spatial cues (Ma et al., 2024). CanKD introduced a cross-attention-based non-local operation for feature-based distillation, achieving superior performance on object detection benchmarks (Sun and Ohyama, 2026). FFKD-Net proposed a hierarchical knowledge distillation module with soft-label supervision and regression consistency constraints for lightweight small-object detection (Chen et al., 2026). CrossKD (Wang J. et al., 2024) addresses the key limitation of traditional prediction mimicking—the conflict between ground-truth supervision and distillation targets—by feeding intermediate features from the student’s detection head into the teacher’s head. The resulting cross-head predictions are then forced to mimic the teacher’s output, relieving the student’s head from contradictory supervision signals. However, the majority of these methods assume that teacher and student operate at the same input resolution—a condition that does not hold in our target scenario where field-portable devices inherently capture low-resolution imagery.

While knowledge distillation has been extensively studied for object detection, most existing approaches focus on model compression within the same input resolution—compressing a large teacher to a small student that shares the same input size. These methods, however, cannot directly address the cross-resolution scenario where the teacher and student operate on fundamentally different input resolutions. A few recent studies have explored cross-resolution distillation for remote sensing (Huang et al., 2025) and low-resolution object recognition (Ren et al., 2025) but they typically rely on complex architectural modifications or additional trainable alignment modules. In contrast, our framework introduces a plug-and-play attention-based distillation loss that operates directly on the neck-level feature maps (P3, P4, P5) with minimal architectural overhead—requiring only a fixed bilinear upsampling layer to resolve spatial mismatches, without introducing any trainable parameters. This design choice ensures that the distillation process does not interfere with the student’s detection head, preserving the original YOLO architecture for efficient deployment. Our approach systematically investigated cross-resolution knowledge distillation for algae detection under field-portable settings, where low-resolution input is a hard constraint rather than an optional trade-off.

## 2 Materials and methods

### 2.1 Dataset

The dataset involved in this study is the publicly available EMDS-7 (Yang et al., 2023) which comprises 2,365 images and 13,216 labeled objects across 42 eukaryotic microorganism (EM) categories. The images were collected from lakes and rivers in Shenyang, China, using a 400 × optical microscope by two environmental biologists between 2018 and 2019. Manual annotation was performed in Pascal VOC format by four bioinformatics scientists from 2020 to 2021, following two rules: fully visible or >60% visible EMs were assigned one of 41 category labels, while <40% visible unknowns, non-listed EMs, and background impurities were marked as ‘Unknown’.” In addition, we also evaluate our method on the publicly available PMID2019 dataset (Han et al., 2019), which comprises 10,819 microscopic images across 24 phytoplankton categories. The images were collected from Jiaozhou Bay, Qingdao, China, using an Olympus BX53 optical microscope at 200 × magnification by marine biologists between 2018 and 2019. Notably, the dataset provides high-resolution color images with instance-level annotations, enabling both object detection and classification tasks.

### 2.2 Method

We propose a knowledge distillation framework for algae detection to transfer representational knowledge from a high-resolution teacher model to a lightweight student model. The overall training objective combines the standard detection loss with a feature-level distillation loss, encouraging the student to mimic the teacher’s internal feature activations.

We adopt YOLO11s as both the teacher and student architectures for three primary reasons. First, using the same architecture eliminates channel-dimensionality mismatches, allowing us to isolate and evaluate the core contribution of cross-resolution distillation without confounding factors from architectural heterogeneity. This design choice ensures that any performance gain can be unambiguously attributed to the resolution distillation itself rather than to architecture-specific alignment tricks. Second, YOLO11s offers a favorable trade-off between detection accuracy and computational efficiency making it suitable as both a high-performance teacher and a lightweight student for field deployment. Third, the shared architecture enables a fair comparison with the undistilled baseline, as the student model is identical in structure to the baseline except for the distillation loss. While heterogeneous teacher-student setups (e.g., YOLOv12x teacher and YOLOv5n student) could potentially yield further gains, they introduce additional variables such as differing receptive fields, feature hierarchies, which complicate the interpretation of distillation effectiveness. We therefore defer heterogeneous distillation to future work and focus on establishing a clear proof-of-concept for cross-resolution attention-based distillation in the homogeneous setting.

**Figure 1:**
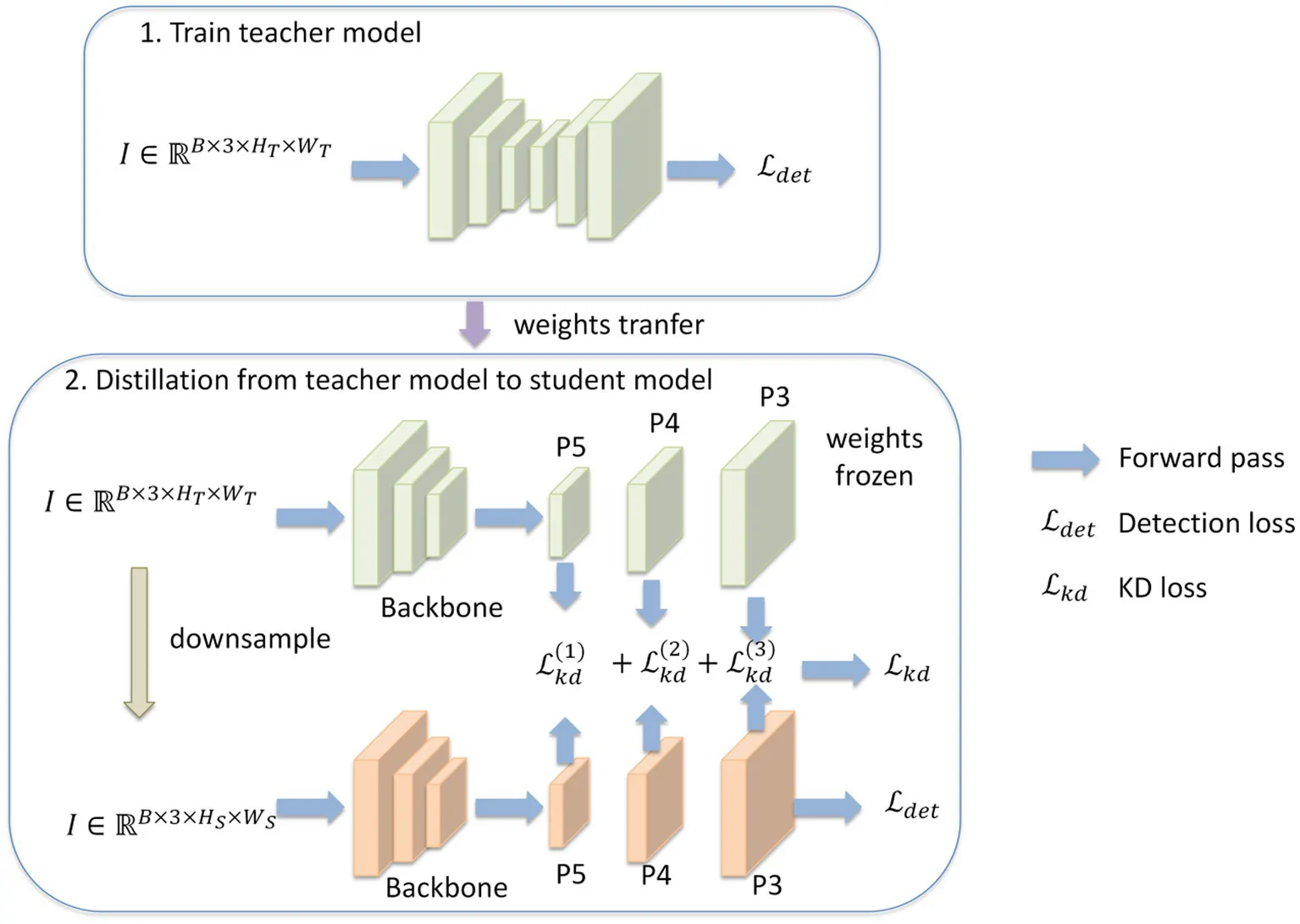
Multi-scale attention-based feature distillation framework.

Let the teacher model T (with frozen parameters) and the student model S (to be trained) have input spatial dimensions $H_{T}\times W_{T}$ and $H_{S}\times W_{S}$ , respectively. Given an input image $I\in {\mathbb{R}}^{3\times H_{T}\times W_{T}}$ at the teacher’s resolution, we first downsample it to a lower resolution $3\times H_{S}\times W_{S}$ using bilinear interpolation to form the student’s input $I_{S}$ . The same original image I is fed to the teacher, while the downsampled version is fed to the student. This design allows the student to operate at a faster inference resolution while still learning from high-resolution teacher cues (Figure 1).

**Figure 2:**
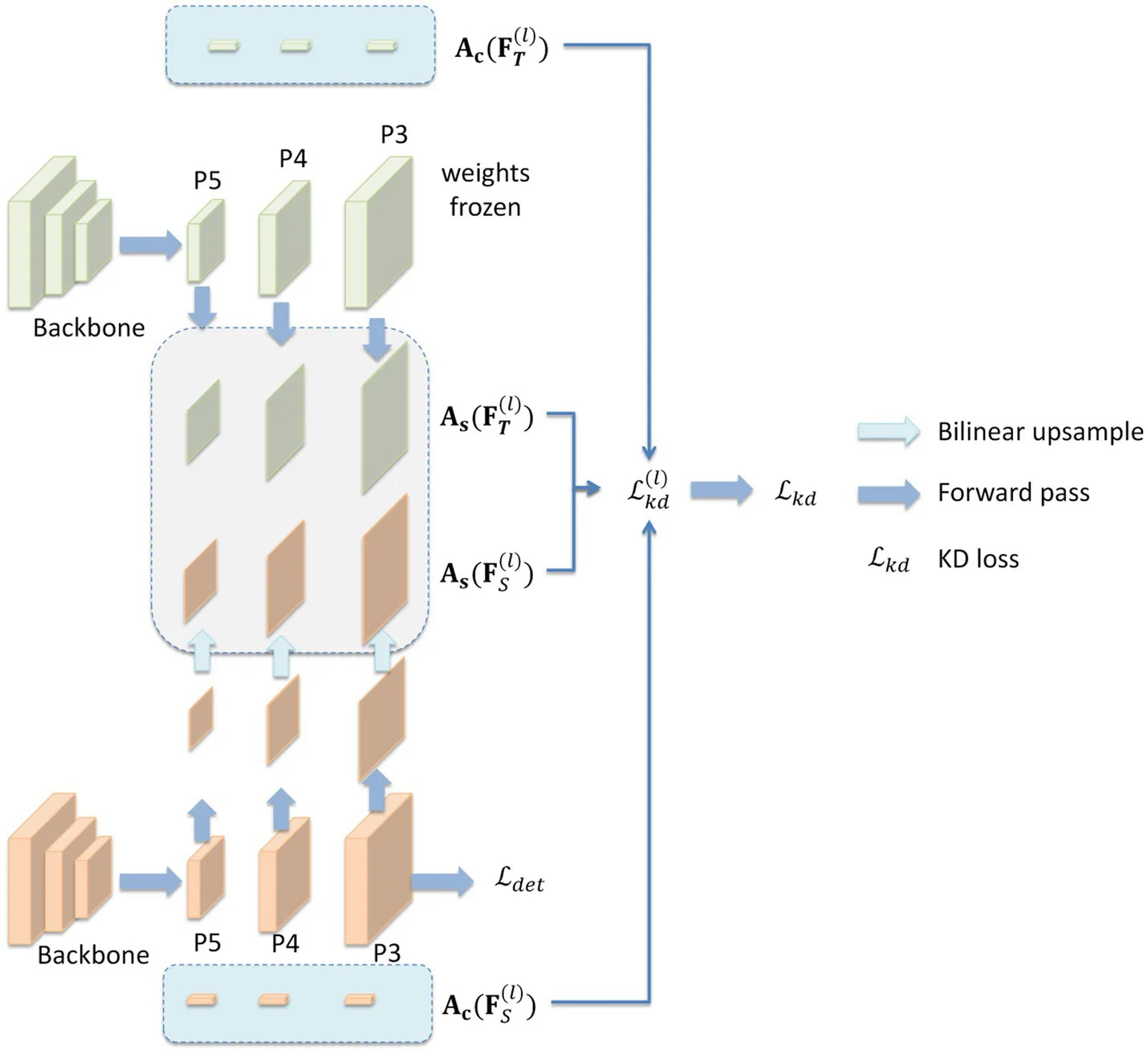
Workflow of distillation loss calculation.

YOLO produce multi-scale feature maps in the neck (e.g., P3, P4, P5) to handle objects of different sizes. Let $F_{S}^{l}\in {\mathbb{R}}^{B\times C_{l}\times H_{l}\times W_{l}}$ and $F_{T}^{l}\in {\mathbb{R}}^{B\times C_{l}\prime \times H_{l}\prime \times W_{l}\prime }$ denote the l -th feature maps produced by the student and the teacher, respectively, where B is the batch size, $C_{l}$ and ${C_{l}}^{\prime }$ are channel numbers, and $H_{l}\times W_{l}$ and ${H_{l}}^{\prime }\times {W_{l}}^{\prime }$ are spatial dimensions at level l . If the spatial dimension differs, we upsample the student’s feature map using a bilinear upsampling layer ${H_{l}}^{\prime }/H_{l}$ (and ${W_{l}}^{\prime }/W_{l}$ ) to match the teacher’s spatial resolution, without introducing trainable parameters. In addition, the channel numbers of $F_{S}^{l}$ and $F_{T}^{l}$ may also be different. However, we use the same architecture for teacher and student in this paper. Therefore, $C_{l}={C_{l}}^{\prime }$ . To avoid forcing exact matching between the feature maps of teacher and student and to focus on where each model “looks,” we adopt an attention-based distillation loss. For a feature map F , we compute its spatial attention map $A_{s}(F)$ and channel attention vector $A_{c}(F)$ as follows:


$$A_{s}(F)=\frac{1}{C}\sum_{c=1}^{C}F{\left(:,c,:,:\right)}^{2}\in {\mathbb{R}}^{B\times 1\times H_{l}\prime \times W_{l}\prime }$$

$$A_{c}(F)=\frac{1}{{H_{l}}^{\prime }{W_{l}}^{\prime }}\sum_{i=1}^{H_{l}\prime }\sum_{j=1}^{W_{l}\prime }F(:,:,i,j)\in {\mathbb{R}}^{B\times C_{l}\prime \times 1\times 1}$$

which highlights regions with high activation energy. The distillation loss for each level l is then the mean squared error (MSE) between the normalized attention maps of the student and the teacher (Figure 2):


$${L_{\mathrm{kd}}^{\left(l\right)}=|\;|A_{s}(F_{S}^{\left(l\right)})-A_{s}(F_{T}^{\left(l\right)})\|}_{2}^{2}+{\left\|A_{c}(F_{S}^{\left(l\right)})-A_{c}(F_{T}^{\left(l\right)})\right\|}_{2}^{2}$$

The total feature distillation loss is summed over the three levels l∈{P3,P4,P5} :


$${ℒ}_{\mathrm{kd}}=\sum_{l}{ℒ}_{\mathrm{kd}}^{\left(l\right)}$$

The overall loss for this framework is:


$$ℒ={ℒ}_{\det}+\lambda {ℒ}_{\mathrm{kd}}$$

where ${ℒ}_{\det}$ is the detection loss (including box regression, objectness, and classification losses); λ is the distillation weight. By enforcing similarity of the attention maps at P3, P4, and P5, the student learns to focus on analogous regions to those attended by the teacher across multiple scales, which is particularly beneficial for detecting objects of varying sizes.

## 3 Experimental results

All experiments are conducted on the EMDS-7 dataset (Yang et al., 2023). The dataset is randomly split into training, validation, and test sets following a 6:2:2 ratio with a fixed random seed of 42, with the split performed on a per-category basis to ensure that each set contains all 42 EM categories. This yields 1,401, 456, and 505 images for the training, validation, and test sets, respectively. All experiments are conducted using this fixed dataset split to ensure fair and consistent evaluation across all methods. Performance is evaluated using the mean Average Precision (mAP) at IoU thresholds from 0.5 to 0.95 (mAP50-95) and mAP50. The mean Average Precision (mAP) is the core evaluation metric for algae detection task which is the mean of the Average Precision of different categories. The precision-recall curve for each category is drawn by varying the confidence threshold and the AP is calculated by computing the AUC of precision-recall curve.


$$\mathrm{AP}=\sum_{k}p(k)\mathrm{\Delta r}(k)$$

where p(k) and r(k) refer to the precision and recall at the k-th operating point, respectively, and Δr(k) is the increment of recall.

The teacher model is a YOLO11s detector trained at a high input resolution of 1,280 × 1,280 pixels, with weights loaded from a pre-trained weights. The student model also follows YOLO11s architecture initialized from COCO-pretrained weights, operating at a reduced resolution of 640 × 640 pixels. Both models share the same output structure, enabling feature-level distillation at the P3, P4 and P5 neck levels.

All models were trained and evaluation on a Windows 11 workstation equipped with an Intel Core i9-14900K processor (3.20 GHz), 64 GB of RAM, and an NVIDIA RTX A5000 GPU, using PyTorch 2.7.0 with CUDA 11.8 and Ultralytics 8.4.9. The initial learning rate, momentum and weight decay follow the default values of the Ultralytics YOLO trainer (e.g., lr = 0.01, momentum = 0.937, weight decay = 0.0005). Input images are preprocessed by first loading the original high-resolution image (1,280 × 1,280) for the teacher, then bilinearly downsampling it to 640 × 640 as the student’s input. No additional data augmentation differences are introduced between the two branches. The training time of our model is 4.35 h. The model contains 9.4 million parameters and incurs 21.6 GFLOPs of computational cost. Inference performance was also evaluation on the Jetson Orin Nano Super 8G device. The results showed that the ONNX-based inference yielded 2.39 FPS and 219.07 MB of RAM usage, whereas the TensorRT-accelerated variant achieved 65.24 FPS, with corresponding GPU and system memory consumptions of 2.05 GB and 1.58 GB, respectively.

The feature distillation loss is applied from the first epoch onwards. The balancing coefficient for the attention-based distillation loss is set to λ=0.5 . For each training batch, the teacher performs a forward pass without gradient computation and with automatic mixed precision (AMP) disabled to ensure numerical stability. Student feature maps at levels P3, P4 and P5 are upsampled when necessary using a fixed bilinear upsampling layer (scale factor 2) to match the spatial dimensions of the teacher’s corresponding feature maps. The attention map for each level is computed as the channel-wise mean of squared activations, followed by an MSE loss.

To ensure a rigorous comparison, we train the baseline model and the model equipped with Multi-scale Attention-based Feature Distillation multiple times using five distinct random seeds. The baseline achieves a mean mAP50-95 of 0.77907 ± 0.00315 (95% CI: [0.775150, 0.782984]), while our proposed method yields 0.78571 ± 0.00221 (95% CI: [0.782966, 0.788455]). The 95% CI for the performance difference is calculated as [0.00445, 0.00884], indicating that the improvement is consistently positive. Furthermore, a paired *t*-test is conducted to evaluate statistical significance, yielding a *p*-value of 0.00109, which demonstrates that the observed performance gain brought by our distillation strategy is statistically significant.

To scrutinize our design choices, we conducted a series of ablation studies. First, we assessed the effectiveness of the proposed attention-based distillation and impact of λ . The results are shown in Table 1. Second, we independently removed the channel and spatial attention modules from the distillation pipeline to quantify their respective contributions. Table 2 summarizes these results, where $A_{c}$ and $A_{s}$ denote the retention of channel and spatial attention, respectively, and $A_{c}+A_{s}$ corresponds to our complete approach. The ablation study in Table 2 demonstrates that incorporating both channel and spatial attention ( $A_{c}+A_{s}$ ) yields the best overall performance across mAP metrics. Third, we investigated the sensitivity of the model to the teacher network’s input resolution, with the findings reported in Table 3. The results showed that a higher input resolution does not always improve detection performance. Although it preserves more spatial details, the fixed receptive field of the model covers a relatively smaller portion of the enlarged image, weakening global context perception. Furthermore, we assessed the individual and combined effects of transfer learning (TL) and knowledge distillation (KD); as demonstrated in Table 4, both techniques consistently improve detection accuracy. In particular, transfer learning (TL) serves as the primary contributor to performance. Finally, we compared our multi-scale distillation strategy against a single-scale counterpart, where feature distillation is performed on only one of the three output levels (P3, P4, or P5) at a time. Table 5 compares single-scale distillation (P3, P4, or P5) against the multi-scale combination. The multi-scale strategy achieves the best overall mAP50-95 (0.78832), outperforming all individual scales. Although single-scale settings each excel in certain metrics (e.g., P3 in precision, P5 in recall), only the multi-scale approach delivers a balanced trade-off, confirming that integrating multiple feature levels consistently improves detection robustness. We therefore adopt the multi-scale scheme.

**Table 1:** Ablation study on weights of distillation loss λ .

λ	mAP50-95	mAP50	Precision	Recall
0	0.78434	0.87341	0.86408	0.84141
0.25	0.78465	0.87041	0.87409	0.83584
0.5	**0.78832**	**0.87897**	**0.87542**	0.84143
0.75	0.78056	0.87305	0.85318	**0.84939**
1	0.77244	0.86574	0.85163	0.83702

Values in bold represent the maximum values in each column.

**Table 2:** Ablation study on the individual and combined effects of channel and spatial attention in the distillation framework.

Setting	mAP50-95	mAP50	Precision	Recall
$A_{c}$	0.78054	0.87132	0.88477	0.84020
$A_{s}$	0.78290	0.86973	**0.89610**	0.82030
$A_{s}+A_{c}$	**0.78832**	**0.87897**	0.87542	**0.84143**

Values in bold represent the maximum values in each column.

**Table 3:** Impact of teacher input resolution on detection performance.

Resolution	mAP50-95	mAP50	Precision	Recall
960	0.78205	0.86852	**0.88258**	0.81820
1,280	**0.78832**	**0.87897**	0.87542	**0.84143**
1,600	0.77524	0.87149	0.87980	0.83629

Values in bold represent the maximum values in each column.

**Table 4:** Ablation study on knowledge distillation and transfer learning (individual vs. combined).

Setting	mAP50-95	mAP50	Precision	Recall
Baseline	0.49916	0.61874	0.66476	0.56910
Baseline+KD	0.52094	0.65544	0.65024	0.59241
Baseline+TL	0.78434	0.87341	0.86408	0.84141
Baseline+KD + TL	**0.78832**	**0.87897**	**0.87542**	**0.84143**

Values in bold represent the maximum values in each column.

**Table 5:** Comparison of single-scale and multi-scale feature distillation across different output levels.

Scale	mAP50-95	mAP50	Precision	Recall
P3	0.78557	**0.88289**	**0.89040**	0.82263
P4	0.78347	0.87732	0.86984	0.83187
P5	0.78140	0.87220	0.88325	**0.84144**
P3 + P4 + P5	**0.78832**	0.87897	0.87542	0.84143

Values in bold represent the maximum values in each column.

**Figure 3:**
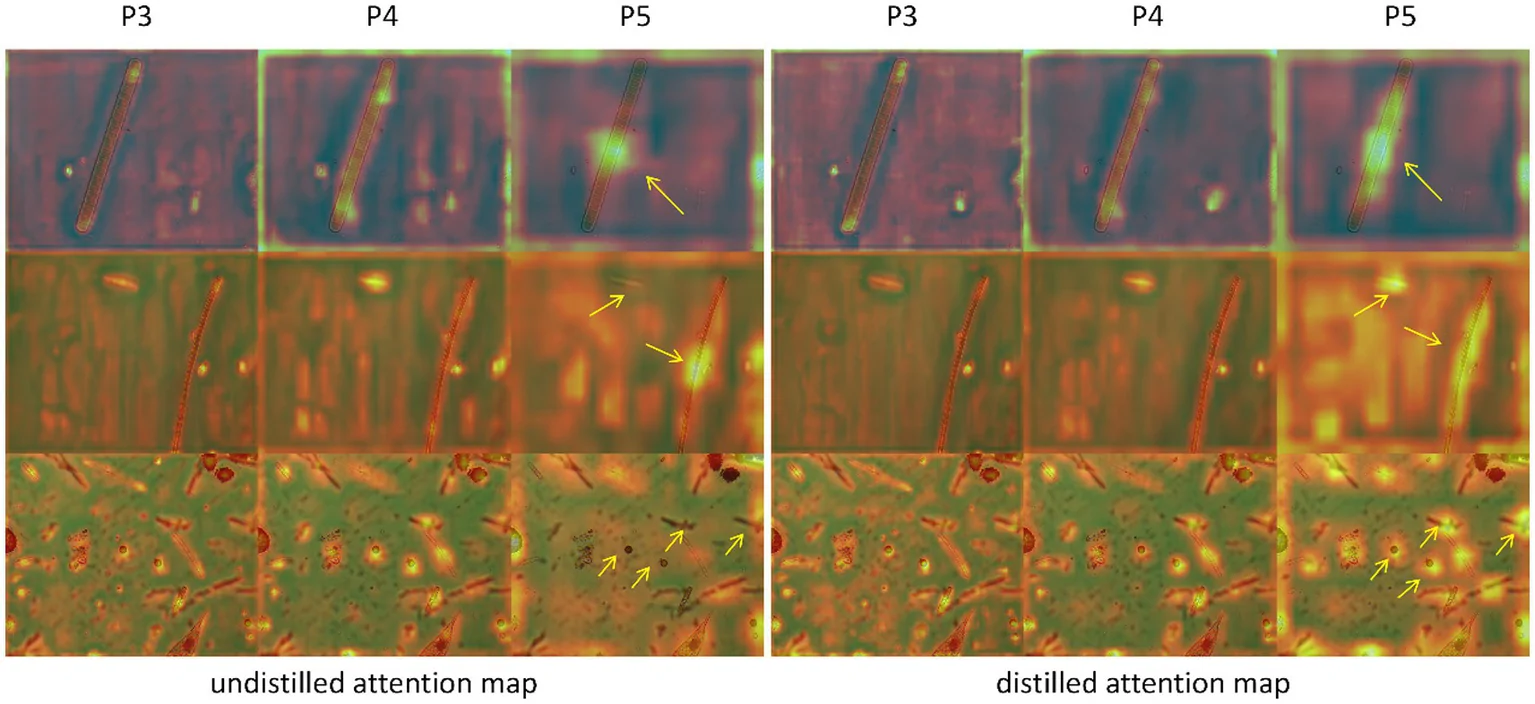
Visualization of attention map generated by undistilled and distilled YOLO11s; The regions indicated by the arrows reveal clear discrepancies.

**Figure 4:**
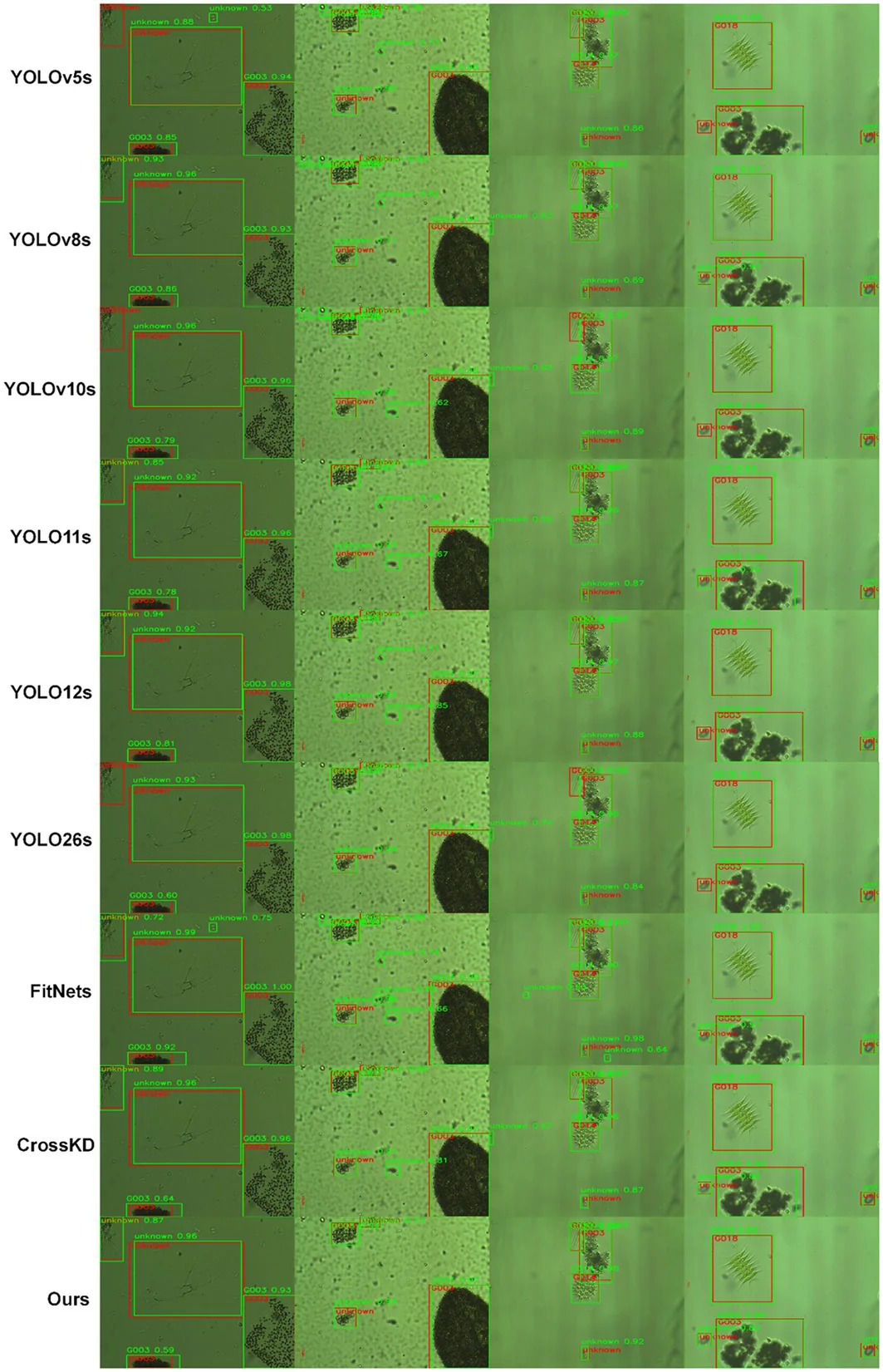
Qualitative comparison of different methods; green and red bounding boxes refer to predictive results and ground truth, respectively.

Furthermore, we visualize the attention map of P3, P4 and P5 generated by undistilled and distilled YOLO11s models in Figure 3. Compared with attention map produced by undistilled YOLO11s, the attention maps generated by distilled YOLO11s exhibit sharper spatial delineation and a more compact foreground focus, yielding visually cleaner and more interpretable heatmaps. As indicated by the arrows, undistilled model exhibits negligible/weak attention responses in these specific regions, whereas distilled model demonstrates stronger and more focused activation (Figure 4).

We compare the proposed attention-based distillation method (denoted as Ours, i.e., YOLO11s + KD) against several state-of-the-art YOLO detectors: YOLOv5s (Khanam and Hussain, 2024a), YOLOv8s (Varghese and Sambath, 2024), YOLOv10s (Wang A. et al., 2024), YOLOv12s (Tian et al., 2026), YOLOv26s (Sapkota et al., 2025), and the undistilled YOLO11s baseline (Khanam and Hussain, 2024b). All models are evaluated on the same test set under identical settings (input resolution 640 × 640 for fair comparison). In addition, we also compared our method with the existed KD strategy [FitNets (Romero et al., 2015) and CrossKD (Wang J. et al., 2024)]. Table 6 summarizes the performance in terms of mAP50-95 and mAP50. In addition, we quantitatively compare the results of methods mentioned in Table 2.

**Table 6:** Comparison with other methods on EMDS-7.

Model	mAP50-95	mAP50	Precision	Recall
YOLOv5s	0.76133	0.86601	0.86908	0.82609
YOLOv8s	0.77689	0.86874	0.87778	0.81903
YOLOv10s	0.78198	0.87482	0.86429	0.84318
YOLO11s	0.78434	0.87341	0.86408	0.84141
YOLO12s	0.77558	0.86640	0.87303	0.83499
YOLO26s	0.77999	0.87488	0.86533	**0.84629**
FitNets	0.74559	0.86078	0.86733	0.82740
CrossKD	0.78206	0.87150	**0.88383**	0.82821
Ours	**0.78832**	**0.87897**	0.87542	0.84143

Values in bold represent the maximum values in each column.

To further validate the advantage of our method, the comparison experiments was also performed on PMID2019 dataset which has 2,725, 2,727 and 5,456 images for train, validation and test set, respectively. The results are shown in Table 7 which also showed that our method achieved the highest mAP50-95 and mAP50.

**Table 7:** Comparison with other methods on PMID-2019.

Model	mAP50-95	mAP50	Precision	Recall
YOLOv5s	0.74211	0.85363	**0.93651**	0.80763
YOLOv8s	0.74879	0.85036	0.92851	0.80687
YOLOv10s	0.75625	0.85614	0.92559	0.80554
YOLO11s	0.75124	0.84752	0.91081	0.81143
YOLO12s	0.76010	0.85349	0.92928	0.81542
YOLO26s	0.76298	0.85477	0.91980	**0.82016**
CrossKD	0.75845	0.85945	0.91383	0.81263
Ours	**0.76808**	**0.87157**	0.93209	0.81553

Values in bold represent the maximum values in each column.

**Figure 5:**
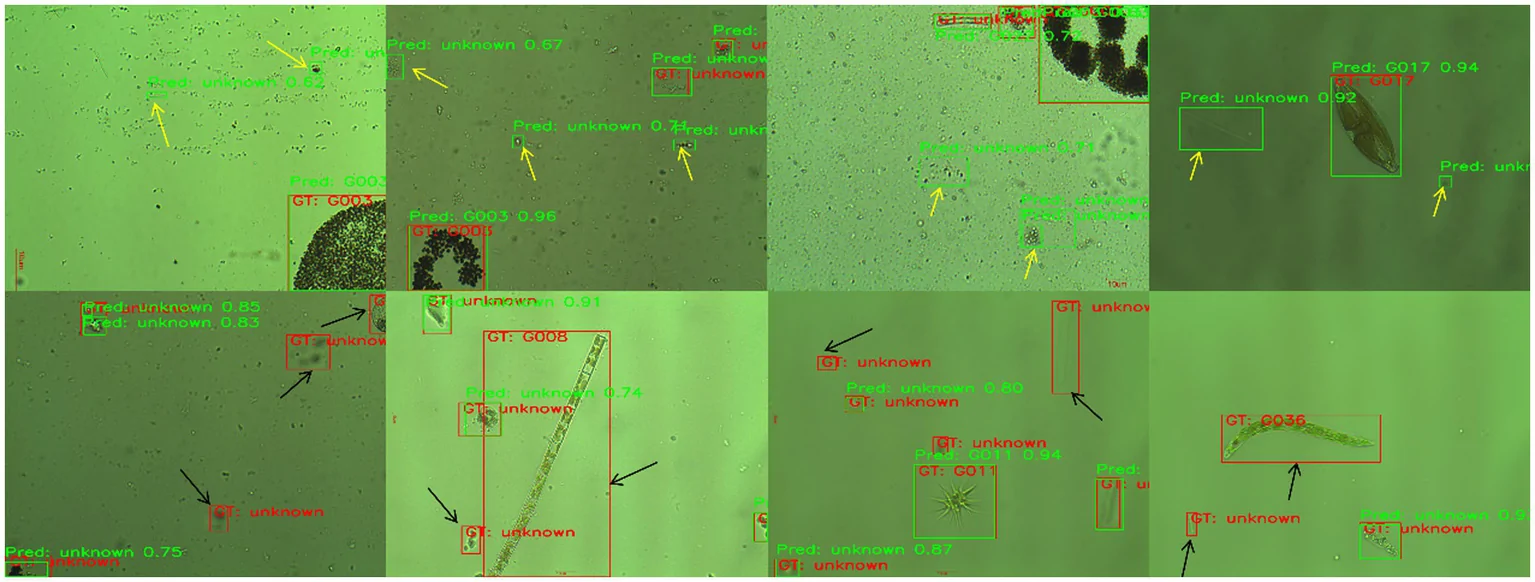
Failure cases of our proposed method where yellow and black arrows indicate false positives and false negatives, respectively.

To complement the quantitative evaluation, we conduct a qualitative error analysis by visualizing typical false positives and false negatives, which reveal the remaining challenging scenarios for our model. The qualitative results are shown in Figure 5.

## 4 Discussion

In this paper, we presented a cross-resolution knowledge distillation framework that transfers fine-grained features from a high-resolution teacher to a low-resolution student via multi-scale attention alignment. The distilled student achieves superior mAP50-95 and mAP50 over the baseline and several YOLO variants, with no extra inference cost—making it suitable for field deployment.

Beyond the specific application of harmful algae monitoring, this work carries broader implications for resource-constrained environmental sensing. The plug-and-play nature of our distillation loss—requiring no trainable alignment parameters—offers a generalizable blueprint for enhancing lightweight detectors across various domains where high-resolution inputs are impractical during deployment, such as underwater robotics, satellite-based monitoring, and mobile health diagnostics. The successful integration of transfer learning with cross-resolution distillation also suggests a promising paradigm for few-shot domain adaptation: a high-resolution teacher pre-trained on abundant data can guide a low-resolution student in data-scarce scenarios, reducing the annotation burden for rare or emerging targets.

Our ablation studies systematically examine four design aspects: distillation strength, attention components, teacher resolution, and multi-scale distillation strategy. The ablation on λ (Table 1) reveals a clear trade-off: λ=0.5 yields the best mAP50-95, while higher values ( λ≥0.5 ) degrade precision despite stable recall, indicating that excessive mimicry of the teacher’s high-resolution feature space introduces false positives in the low-resolution domain. Complementary to this, the attention ablation (Table 2) shows that spatial attention alone ( $A_{s}$ ) achieves higher precision but lower recall, whereas channel attention alone ( $A_{c}$ ) provides more balanced recall. Their combination ( $A_{s}+A_{c}$ ) delivers the best overall mAP50-95, suggesting that spatial attention helps localize discriminative regions while channel attention preserves feature selectivity—a synergy that is particularly beneficial when the student operates at reduced resolution. Counter-intuitively, Table 3 shows that increasing the teacher’s input resolution from 1,280 to 1,600 degrades mAP50-95. We attribute this to the fixed receptive field. The effective receptive field covers a proportionally smaller area of the enlarged image, weakening global context perception for large algae cells. This finding suggests that excessively high teacher resolution does not guarantee better distillation and that 1,280 × 1,280 strikes the optimal balance between spatial detail and contextual integrity. Table 4 further underscores the dominant role of transfer learning (TL): TL alone raises mAP50-95 dramatically, while adding KD on top yields a marginal but consistent improvement to 0.78832. This indicates that cross-resolution distillation is most effective when built upon a strong visual backbone, rather than serving as a standalone remedy for data scarcity. Table 5 demonstrates that single-scale distillation—whether at P3, P4, or P5—optimizes specific metrics (e.g., P3 excels in precision, P5 in recall) but fails to deliver a balanced trade-off. Only the multi-scale combination achieves the best overall mAP50-95, confirming that aligning attention maps across all three pyramid levels is a better strategy than single-scale alignment.

Despite the promising results, several limitations remain. First, failure qualitative analysis reveals that both extremely small and large objects frequently incur false negatives and false positives. Small instances suffer from detail loss and background confusion, while large ones exceed the receptive field, leading to partial misclassification and missed detections. These scale-induced errors highlight the need for more adaptive feature extraction. Additionally, our experiments are conducted on EMDS-7 and PMID2019, which focus on environmental microorganisms under controlled conditions; generalizability to other species or field images with illumination variation and debris interference requires further validation. Finally, the teacher and student share the same architecture to simplify feature-level matching; whether the framework extends to heterogeneous architectures remains an open question.

## 5 Conclusion

This paper presents a cross-resolution knowledge distillation framework that compensates for fine-grained information loss in low-resolution algae microscopy via high-resolution teacher supervision and multi-scale attention alignment. The distilled student achieves superior mAP50-95 and mAP50 over the baseline and several YOLO variants, at no extra inference cost. Beyond algae monitoring, the plug-and-play nature of our distillation loss offers a generalizable strategy for enhancing lightweight detectors in resource-constrained scenarios such as underwater robotics and satellite-based sensing. Future work includes extending to heterogeneous architectures, incorporating self-supervised pre-training, and validating under diverse field conditions.

## Data Availability

The original contributions presented in the study are included in the article/supplementary material, further inquiries can be directed to the corresponding author.
